# The Montreal Veterinary College

**Published:** 1887-04

**Authors:** 


### THE MONTREAL VETERINARY COLLEGE.

The Montreal Veterinary College and the Council of Agriculture celebrated the closing of the session for 1887. There was a large attendance of the pupils and their friends.

Mr. Blackwood, chairman of the agricultural committee of the Council of Arts and Agriculture, presided. There were also present Prof. McEachran, Dr. Leclerc, secretary Council of Agriculture; Mr. Casgrain, Dr. Brydon, of Boston, and Dr. Gadsden, of Philadelphia.

The students passed examinations in order of merit as follows:

Botany (D. P. Penhallow, M. A., professor)—Darling, Harris, Chapin, Vannest, Bateman, Austin, McWhinnie, Wieland, York, Simpson, Parker, Dillon, Harwood, Henderson, Mullins, Meldrum, Goddard and McCurdy.

Histology (Geo. Wilkins, M. D., professor)—Pease, Macaulay, Harwood, Baterman, York, Miller J., McCurdy, McWhinnie, Paige, Vannest, Wieland, Austin, Darling, Chapin, Harris, Simpson M., Skaife, Munro, Goddard and Henderson.

Chemistry (G. P. Girdwood, M. D., professor)—Pease, Miller J., Dawes, Macaulay, Feron, Paige, Robertson, Munro and McGarth.

Physiology (T. Wesley Mills, M. D., professor)—Torrance, McGarth, Becket, Paige, Robertson, Roberts, Miller J., Vannest, Baker, Smith, Muuro and Dawes.

Materia Medica (James Stewart, M. D., professor)—Paige, Munro, Becket, Pease, McGarth, Smith, Vannest, Macaulay, Dawes, Meldrum, Roberts and Robertson.

Cattle Pathology (Charles McEachran, V. S., professor)—Torrance, Rowat, Miller F., Simpson C. R., Feron, Murphy, Baker and Craig.

Anatomy (Mr. C. Baker, V. S., professor)—Torrance, Rowat, Miller F., Feron, Murphy and Simpson C. R., equal, and Baker.

General Pathology, practice of Veterinary Medicine and Surgery (D. McEachran, F. R. C. V. S., professor)—Torrance, Rowat, Feron, Miller, F., Simpson C. R., Baker and Metcalf.

Final oral examination by the board of examiners appointed by the Council of Agriculture, consisting of Messrs. J. W. Gadsden, M. R. C. V. S., of Philadelphia; Williamson Bryden, V. S., Boston; J. A. Couture, V. S., Quebec; Arch. McCormick, V. S., Ormstown, Que.; A. W. Harris, V. S., Ottawa; Geo. Leclerc, M. D. The following students passed: Messrs. Torrance, Rowat, Miller, Feron, Simpson C. R., and Baker.

The diplomas and prizes were distributed by Dr. Leclerc. The following gentlemen having attended the prescribed three sessions and passed the written and oral examinations in botany, physics, histology, chemistry, physiology, materia medica, anatomy, cattle pathology, general pathology and practice of veterinary medicine and surgery, received the diploma, viz.: Messrs. Torrance, Rowat, F. Miller, Feron, Simpson and Baker.

For the best general examination in all subjects, a silver medal, the gift of the Council of Agriculture, won by W. J. Torrance. Practice of veterinary medicine and surgery—1st, W. J. Torrance; 2d, A. R. Rowat. Cattle pathology, W. J. Torrance. Anatomy, W. J. Torrance.

Practice of veterinary medicine and surgery—1st, H. R. Macaulay; 2d, John Miller. Cattle pathology, Jas. B. Paige and H. R. Macaulay, equal. Anatomy, J. F. Pease. Materia medica—1st, Jas. B. Paige; 2d, M. Munro.

Dr. Brydon congratulated the Montreal Veterinary College on its high standing among the veterinary schools. "Whatever," he said, "might be gained by rushing men through a school years ago, there was nothing to be gained now, as the profession was well enough supplied with members in Canada."

Dr. McEachran, in the course of his speech, said that since the establishment of the school in 1866 it had made great progress, and its connection with McGill University gave it a position which it could not otherwise attain. In undertaking the establishment of the school he knew he would have great responsibility and he looked forward to an uphill fight. The years which had passed confirmed him in that opinion, but he meant to aim high in veterinary education and he succeeded (Applause). They had established a matriculation examination in their school and given a thorough scientific course which they might be proud of when they found young men like Messrs. Pease and Torrance taking as large a per centage as any of the medical students. In Toronto there was no matriculation needed, and when intending students saw by the course that they had to pass this examination in Montreal, it prevented one-half of them coming, and another one-third were prevented when they found they would have to pass three sessions at the school. His policy was that the national school was more honored by turning out one man who would be a credit to it than one hundred semi-illiterate men who would not be an honor to any profession.

After a vote of thanks had been proposed to the chairman the proceedings terminated.

The last meeting of the Veterinary Medical Association session was held in the college lecture room at 5 P. M., the president, Mr. M. C. Baker, V. S., in the chair.

Mr. G. W. Becket was elected secretary-treasurer and librarian for the summer months.

The following members having fulfilled the necessary requirements received the diploma of the association: Torrance, Simpson, Rowat, Feron, Miller and Baker.

Prof. T. Wesley Mills made the report of the committee appointed to examine the essays and awarded the prize, a valuable case of surgical instruments, to Mr. Torrance, the committee deciding that his was the best paper read during the session. He (Prof. Mills) said that he regretted there was not another prize, as he considered Mr. Simpson's paper to have been an exceptionally good one, and worthy of a second prize. In fact all the papers were of a very high order of merit, and were distinguished by a degree of originality not often seen in students' papers.

